# One Social Media, Distinct Habitus: Generation Z's Social Media Uses and Gratifications and the Moderation Effect of Economic Capital

**DOI:** 10.3389/fpsyg.2022.939128

**Published:** 2022-07-13

**Authors:** Qingqing Hu, Xue Hu, Pan Hou

**Affiliations:** School of Journalism and Communication, Northwest University, Xi'an, China

**Keywords:** generation Z, social media, uses and gratifications, economic capital, habitus

## Abstract

This study aims at contributing to literature by investigating characteristics of Generation Z's social media uses and gratifications and the moderation effect of economic capital. Specifically, we employed online survey as the main research method to examine the connections between the young generation cohort's online motivations, social media practices, and economic capital. A total of 221 Chinese Generation Z social media users were recruited in the survey. Results indicated that (1) Generation Zs have different social media engagements depending on whether they were connected for daily routine alternatives or socialization; (2) the young cohorts from upper-mid-income families demonstrated a more instrumental-rational habitus to use social media more frequently as a communicative tool than those from low-income families; and (3) motivations and family income interacted to influence Generation Z's social media practices (e.g., social capital accumulating and exchanging and self-expression). Findings here provide empirical reference to deepened understandings of the interactions between social media and digital generations, and their connections with digital social inequalities.

## Introduction

Generation Z (Gen-Z) refers to people born between the mid-1990's and 2009, who grew up with the digital society and view digital technologies as the foundation of their lives (Turner, 2015; Dimock, 2019). Gen-Z belongs to a larger social media user population that has been conceptualized as the digital natives (Prensky, 2001). Digital natives were born and raised in the digital age, and they spend most of their lives surrounded by and using computers, video games, cell phones, and all the other toys and tools of the digital age (Prensky, 2001). While the rationality of the conceptualization of digital natives is still in debate, a growing number of research has indicated that the younger generations significantly differ from their predecessors in terms of technology-related perceptions, motivations, and behaviors (Hargittai, 2010; Curtis et al., 2019; Hu and Cheong, 2021).

China has ~300 million Gen-Z and 30 million Gen-Alpha (i.e., those born in 2010–2024) Internet users, constituting 1/3 of China's Internet user population (China Internet Network Information Center, 2021). Most (99.2%) of China's internet users are also social media users, and about 1/4 of them spend more than 4 h/day on WeChat, one of China's most popular social media (An, 2021; China Internet Network Information Center, 2021). Previous studies have discussed from multiple perspectives the predictors, characteristics, mechanisms, and consequences of the social media-user interactions in China (e.g., Sullivan, 2012; Wang et al., 2015; Chen et al., 2016; Gan et al., 2017; Li et al., 2019). However, what has been relatively understudied is the extent to which Chinese Gen-Zs' social media habitus are associated with their characteristics as being digital natives, as well as how their social media engagements are associated with digital social inequalities (Gentina, 2020; Hu and Cheong, 2021). In this study, therefore, the main objective is to fill these gaps by examining the characteristics of and differences in social media habitus among Chinese Gen-Z users, as well as exploring how their social media uses and gratifications are associated with economic capital using the Bourdieusian approach (Ignatow and Robinson, 2017; Calderon Gomez, 2021).

## Literature Review

One influential theoretical framework to approach the social media-user interactions is the uses and gratifications theory (U&G), which highlights the importance of individuals' social and psychological needs in shaping their motivations and, consequently, their communicative behaviors (Katz et al., 1973; Rubin, 1994; Papacharissi and Rubin, 2000; Abbas and Mesch, 2015). On one hand, previous studies indicated that Gen-Zs differ from their predecessors in many aspects of online motivations, such as being more desired for self-expressing and self-disclosing, online shopping, online enjoyment, memetic engagements, content-generating, and sustainable online behaviors (Hargittai, 2010; Turner, 2015; PrakashYadav and Rai, 2017; Dabija and Lung, 2018; Dabija and Bǎbuţ, 2019; Viţelar, 2019; Andronie et al., 2021; Hu and Cheong, 2021; Musova et al., 2021; Vǎtǎmǎnescu et al., 2021). On the other hand, the extent to which social media uses can satisfy Gen-Zs' online motivations depends on their affordances. Social media affordances keep evolving with the development of the technologies and the industry, shifting from a focus on networked communication to the scope of online sociality (Boyd and Ellison, 2007; Zhang and Pentina, 2012; Van Dijck, 2013; Choi et al., 2020). There have been discussions on how socialization, as a pivotal social media affordance, plays an important role in satisfying some of Gen-Zs' online motivations (e.g., enhancing social influence and increasing social capital, Shane-Simpson et al., 2018; Andronie et al., 2021). In this study, we will contribute to the discussions by exploring how Gen-Zs' social media uses, including both socialization and beyond, connect with their online motivations in the Chinese context. Therefore, we propose our first research question.

*RQ*_1_: What are the relationships between Chinese Gen-Zs' online motivations and social media uses?

Previous studies indicated that individuals' technological engagements are associated with inequalities in their social, economic, and cultural status and life opportunities (Norris, 2001; DiMaggio et al., 2004; Van Dijk, 2006; Zillien and Hargittai, 2009; Mossberger et al., 2012). In light of the limitations of a functionalist perspective to studying digital social inequalities, scholars introduced the Bourdieusian approach that views technological engagements as occurring in social spaces made up of interrelated fields constraining and shaping each other, with distinctive user habitus and capital (Halford and Savage, 2010; Ignatow and Robinson, 2017; Hu and Cheong, 2021). According to Bourdieu (1984, 1986), habitus is a set of dispositions that structures individuals' practices, and capital refers to socially valued assets (e.g., economic wealth, social relations, and cultural resources) that can influence individuals' status in the system through accumulating and exchanging.

The conceptualizations of habitus and capital contribute to deepened understandings of Gen-Zs' social media uses and gratifications. Social media habitus serves as an embodiment of the interactions between Gen-Zs and their situated socioeconomic context, and it shapes and repeatedly magnifies user disparities in social media practices through machine learning and algorithms (Hu and Cheong, 2021; Hopkins, 2022; Kliestik et al., 2022a,b; Nica et al., 2022). Capital is another key to understanding the predictors and consequences of Gen-Zs' social media practices (Ignatow and Robinson, 2017; Calderon Gomez, 2021). Previous research showed significant correlations between economic capital and technology-related habitus, which further connect with digital social inequalities (e.g., Robinson, 2009). As for Gen-Z, there is still insufficient knowledge to unpack how the youth from low- and upper-mid-income families would differ in terms of their preference of social media practices, and how economic capital can moderate their social media uses and gratifications. Hence, we propose the second and third research questions.

*RQ*_2_: What are the differences in social media practices between Gen-Zs from low- and upper-mid-income families?

*RQ*_3_: How does economic capital moderate Gen-Zs' social media uses and gratifications?

## Methods

### Procedures and Participants

This study employed an online survey for data collection. The questionnaire was adapted from literature and revised based on several pilot studies (van Teijlingen and Hundley, 2002). Participants were students from a large public university in Southwestern China. Participants consisted of 221 Chinese Gen-Z social media users (male = 89, female = 132), and aged between 20 and 24 (*M* = 22.29, *SD* = 1.13). Annual family income was coded as low (*n* = 96, 43.4%) and upper-mid (*n* = 125, 56.6%) using ¥50,001–¥100,000 (≈$7,455–$14,910) as the threshold (China Bureau of Statistics, 2018).

### Measurements

#### Online Motivations

Ten items were adapted from literature (e.g., Papacharissi and Rubin, 2000; Liu and Li, 2010; Turner, 2015; China Internet Network Information Center, 2017; Andronie et al., 2021), and were measured on a 5-point Likert scale (1 = “strongly disagree,” 5 = “strongly agree”). A principal component factor analysis identified two dimensions of the motivations (53% explained variance). Daily routine alternatives were motivations regarding gaining information (0.83), entertainment (0.80), online shopping (0.61), and doing school- and work-related things (0.70); socialization included seeking help (0.78), sharing with others (0.62), developing/maintaining relationships (0.60), and self-promotion (0.74).

#### Social Media Use

Nineteen items (1–7, 1 = never, 7 = more than three times a day) measured how frequent participants engaged in social media activities. A principal component factor analysis constructed four dimensions (60% explained variance): networked communication (NC) included checking updates (0.76), liking/commenting (0.78), communicating with others (0.67), and checking group-discussion records (0.62); social capital accumulating and exchanging (SCAE) included asking for help (0.74), supporting others (0.77), self-promotion (0.62), and obtaining self-beneficial information (0.63); self-expression (SE) included posting updates (0.69), sharing selfies (0.80), and self-expressing (0.67); news watching (NW) included watching news *via* social media (0.84).

#### Demographics

Participants' age, gender, education background, urbanness, and annual family income were collected.

## Results

**Figure 1:**
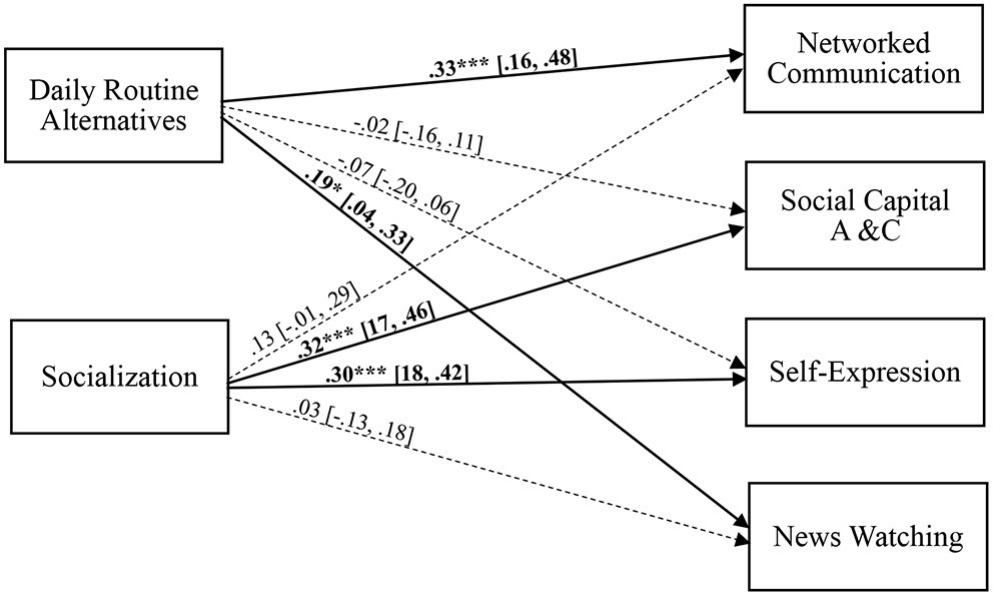
The full model with standardized estimates. *p < 0.05. ***p < 0.001. The solid line represents significant effects. Ninety-five percent confidence intervals are reported within brackets. Model fit statistics: χ^2^ = 0.16, *df* = 1, *p* = 0.69, CFI = 1.00, TLI = 1.09, RFI = 0.99, NFI = 1.00, RMSEA = 0.00.

For *RQ*_1_, we used SEM to explore the relationships between Gen-Zs' online motivations and social media uses, with demographics as covariates. A bootstrapping technique with 5,000 replicates was performed to achieve generalizability beyond the sample. Results indicated that the daily routine alternatives motivation significantly predicted NC and NW, and the socialization motivation was associated with SCAE and SE on social media (see Figure 1).

For *RQ*_2_, significant differences in social media practices were observed between low- and upper-mid-income families, when controlling for age, gender, and education background. Gen-Zs from upper-mid-income families used social media for more NC [*F*_(1,216)_ = 17.78, *p* < 0.001] and NW [*F*_(1,216)_ = 5.01, *p* < 0.05] than those from low-income families, whereas two groups did not significantly differ in SCAE and SE on social media.

Regarding, *RQ*_3_, several hierarchical regressions (block 1 = covariates; block 2 = motivations and income, block 3 = interaction effects) were conducted. For Gen-Zs with the daily routine alternatives motivation, income significantly moderated NC, *B* = −0.23, β = −1.12, *t* = −2.67, *p* < 0.01. For those driven by the socialization motivation, income significantly moderated SCAE (*B* = 0.29, β = 1.26, *t* = 3.12, *p* < 0.01) and SE (*B* = 0.17, β =0.81, *t* = 1.93, *p* < 0.05). We used Dawson and Richter's (2006) approach to probe the interaction effects. As daily routine alternatives motivation became stronger, the low-income participants had a greater increase of their NC on social media (from 4.91 to 5.91) than those with upper-mid-income (from 5.80 to 6.13). When socialization motivation was low, Gen-Zs with more economic capital showed less SCAE (*M*_*low*_ = 6.00, *M*_*upper*−*mid*_ = 5.69) and SE (*M*_*low*_ = 4.32, *M*_*upper*−*mid*_ = 4.23) than those from low-income families; whereas when the richer were strongly motivated to socialize online, they would surpass the poorer in both social media practices (SCAE: *M*_*low*_ = 6.23, *M*_*upper*−*mid*_ = 6.78; SE: *M*_*low*_ = 4.69, *M*_*upper*−*mid*_ = 5.15).

## Discussion

Results indicated that Chinese Gen-Zs have different social media uses depending on two categories of online motivations: social media as communicative tools and news portals when they are doing their daily routines online; and as platforms for social capital accumulating and exchanging and self-expression during online socialization. The findings are consistent with and extending literature on characteristics of digital natives (e.g., Turner, 2015; Viţelar, 2019) and indicate different social media uses based on different scenarios. Furthermore, compared to other U&G studies on social media usage (e.g., Turner, 2015; PrakashYadav and Rai, 2017), our results highlighted an emphasis on social capital throughout social media uses and gratifications, and the integration of social media into daily routines by Chinese Gen-Zs.

Another contribution of this study is that we employed Bourdieusian approach to explore how economic capital influences Gen-Zs' social media uses and gratifications. We observed distinct social media habitus between Gen-Zs from low- and upper-mid-income families: the latter embrace a more instrumental-rational habitus to use social media more frequently as a communicative tool; whereas the former value the importance of online socialization to increase their social capital, but have no more practices in related social media activities. Finally, Gen-Zs from upper-mid-income families take a more conservative stance in SCAE and SE when their socialization motivation is low. This finding is consistent with the literature (e.g., Robinson, 2009; Perrin, 2015) that a higher income is not necessarily associated with more frequent social media practices.

## Conclusion

In this study, we examined Chinese Gen-Zs' social media uses and gratifications and found: (1) daily routine alternatives motivation predicts NC and NW, and socialization motivation is associated with SCAE and SE; (2) Gen-Zs from upper-mid-income families employ a more instrumental-rational habitus to use social media as a communicative tool than those from low-income families; (3) Gen-Zs with higher economic capital tend to be more conservative in SCAE and SE when socialization motivation is low. Applying the Bourdieusian approach to U&G studies, this study highlighted the importance of economic capital in Gen-Z's social media practices: it helps formulate distinct social media habitus that may be repeatedly consolidated by machine learning and algorithms, as well as influences social capital accumulating and exchanging; both may lead to more digital social inequalities among Gen-Zs. Despite the limitations (e.g., sample representativity, inclusiveness of motivations and uses), our findings shed light on future studies on connections between economic capital, social media U&G, and digital social inequalities among and across digital generations.

## Data Availability Statement

The raw data supporting the conclusions of this article will be made available by the authors, without undue reservation.

## Author Contributions

QH was in charge of collecting data and writing manuscript. XH did data analysis and reference check. PH did results report and figure. All authors contributed toward the manuscript.

## Funding

This work was funded by National Social Science Funding of China, Number: 19BXW060.

## Conflict of Interest

The authors declare that the research was conducted in the absence of any commercial or financial relationships that could be construed as a potential conflict of interest.

## Publisher's Note

All claims expressed in this article are solely those of the authors and do not necessarily represent those of their affiliated organizations, or those of the publisher, the editors and the reviewers. Any product that may be evaluated in this article, or claim that may be made by its manufacturer, is not guaranteed or endorsed by the publisher.
